# Structuring Broiler Barns: How a Perforated Flooring System Affects Animal Behavior

**DOI:** 10.3390/ani12060735

**Published:** 2022-03-15

**Authors:** Franziska May, Jenny Stracke, Sophia Heitmann, Carolin Adler, Alica Krasny, Nicole Kemper, Birgit Spindler

**Affiliations:** 1 Institute for Animal Hygiene, Animal Welfare and Farm Animal Behavior, University of Veterinary Medicine Hannover, Foundation, 30173 Hannover, Germany; alica.krasny@tiho-hannover.de (A.K.); nicole.kemper@tiho-hannover.de (N.K.); birgit.spindler@tiho-hannover.de (B.S.); 2 Institute of Animal Science, Ethology, University of Bonn, 53115 Bonn, Germany; jenny.stracke@itw.uni-bonn.de; 3 Public Veterinary Service, Animal Health and Animal Husbandry, Schleswig-Holstein, 23795 Bad Segeberg, Germany; s.heitmann@segeberg.de; 4 Department of Animal and Poultry Science, University of Saskatchewan, Saskatoon, SK S7N 5A8, Canada; carolin.adler@usask.ca

**Keywords:** broiler, behavior, perforated floor, elevated platform, animal welfare

## Abstract

**Simple Summary:**

Broiler chickens in Europe are usually raised in littered barns without structuring elements. Previous studies have found a positive influence on the health and welfare of broiler chickens when they have access to elevated platforms. This study aimed to evaluate the effect of an elevated perforated floor on the behavior of broiler chickens. Therefore, one of two barns was equipped with a perforated floor under the food and water supply. The second barn was used as a control. In total, three fattening periods were observed, with 500 broiler chickens kept in each barn. To compare the behavior of the birds, cameras were installed in both barns. The videos were analyzed by counting the number of birds in a defined area and observing focal animals continuously while recording their behavior. More animals were observed on the perforated floor than in the littered control area, but, in total, the focal animals spent less time on the perforated floor compared to the observed littered area in the control barn. There were no differences in the length of the recorded behaviors between the treatments. These findings suggest that, in general, the elevated perforated floor is attractive for the animals. However, it does not promote one of the recorded behavior patterns. Our results show that an elevated perforated floor could be an option for structuring broiler barns.

**Abstract:**

Broiler chickens in Europe are usually raised in a barren environment. Elevated perforated platforms address this problem and can positively influence animal health and welfare. To evaluate the effect of an elevated perforated floor on the behavior of broiler chickens, one of two barns was equipped with a perforated flooring system under the food and water supply. The second barn was used as a control. In total, three fattening periods were observed, with 500 broiler chickens (Ross 308 breed) kept in each barn. To compare the behavior of the birds in these groups, cameras were installed in the two barns. The videos were analyzed by counting the number of birds and observing focal animals while recording their behavior. More animals were observed on the perforated floor than in the littered control area (*p* < 0.001), but focal animals spent less time on the perforated floor compared to the observed littered area in the control barn (*p* < 0.05). There were no differences in the length of the recorded behaviors between the treatments. These findings suggest that, in general, the elevated perforated floor is attractive for the animals. However, it does not promote one of the recorded behavior patterns. Our results show that an elevated perforated floor could be an option for structuring broiler barns.

## 1. Introduction

According to council directive 2007/43/EC, fast-growing broiler chickens in Europe are usually kept on littered concrete floors. This results in barren environments on many farms, providing no structural elements besides feeding and water lines. This lack of environmental complexity is a concern of animal welfare [1], since broilers are not able to perform highly motivated behavior patterns such as perching, which is presumed to lead to frustration and a negative emotional state [2,3]. Further, a barren environment provides no stimuli for activity, which can be one factor for health problems such as leg abnormalities [4]. Providing elevated platforms can be an option to address this problem. They can encourage higher activity [5] and positively impact leg health [6]. Moreover, separating the animals from manure using elevated platforms with a perforated surface has been reported to improve foot pad health [7,8,9]. A pioneer in this field is Switzerland, providing access to elevated platforms to over 90% of broiler chickens by raising them under the animal welfare label ‘BTS’ [10]. The Dutch ‘Better Life’ label also considers elevated structures, for example, straw bales, as crucial [11].

Like their ancestor, the red jungle fowl, domestic chickens are highly motivated to perch on elevated structures such as branches [12]. In husbandry systems for laying hens, elevated structures are an integral part. It is known that the lack of perches leads to frustration in laying hens and has been suggested to reduce animal welfare [2]. Previous research has demonstrated that broilers are also motivated to perch when given the opportunity. However, they prefer elevated platforms to perches [13,14,15], which might be due to their relatively greater body weight [14,15]. Laying hens always choose the highest structure available for perching [16]. Interestingly, Malchow et al. [15] found that mixed-sex fast-growing-broiler chickens at the end of the fattening period also preferred the highest platform tested in their study, which was 50 cm high. However, in the following study, Malchow et al. [5] reported that fast-growing male broilers were observed more often on the lowest level of the platforms (10 cm), presumably because they were heavier than the chickens in their earlier study. A higher body weight, lower leg health, and therefore a decline in activity are suggested to cause a decrease in perching [5,13,14]. It has been observed that installing ramps at an angle of about 15°–35° is a possibility to adapt elevated structures to the needs of broiler chickens [13,14,15,17]. Younger birds also benefit from ramps that allow them to perch [14]. Broiler chickens are motivated to use elevated structures from the first week on [14,15]. An increase in the use of elevated platforms by broiler chickens over the fattening period was found by Bailie et al. [14], with a peak during week five. Afterward, the use of platforms declines presumably because the broilers are larger and need more space and due to their decreasing physical abilities [13,14]. 

An approach for implementing elevated perforated floors into broiler husbandry is a partially perforated flooring system. The installation of a perforated floor beneath the feeding and the water lines could be useful to control the drainage of water lines and could, therefore, improve litter quality [18]. At the same time, the system allows the animals to sit elevated and use the littered area for pecking, scratching, and dustbathing. Previous studies evaluated the effect of a partially perforated flooring system on animal welfare [8,9], production performance [8,9], litter quality [18,19], ammonia emission [20], and antimicrobial resistance [21]. However, the behavior of broiler chickens in this system has not yet been investigated [9,18]. 

This study aimed to evaluate the use of an elevated perforated floor equipped with food and water supply by broiler chickens. We assessed the number of animals on the floor and their time spent on it in the different phases of the fattening period. We predicted that there would be a higher number of animals on the perforated floor and that they would spend more time on it than in a littered control area. We evaluated whether the animals used the elevated floor apart from using the resources. More specifically, we expected that the birds would spend more time either with locomotion or sitting inactive on the perforated floor than in a littered control area. Further, we assessed whether the animals sat inactive for longer periods on the perforated floor. We predicted that the number of animals in both observed areas would decrease and that the duration of sitting inactive would increase over the fattening period. 

## 2. Materials and Methods

### 2.1. Birds and Housing

The study was conducted in two similarly constructed broiler barns at the research farm of the University of Bonn (Königswinter, Germany). Five-hundred fast-growing broiler chickens of the breed Ross 308 (BWE-Brüterei Weser-Ems GmbH & Co. KG, Rechterfeld, Germany) were kept in each barn for 32 days over three non-consecutive trials (1000 animals per trial). The barns had a floor space of 5.3 m × 4.7 m each, which led to a stocking density of 39 kg/m² at the end of the fattening period. This corresponds to 19.5 animals per square meter at a final body weight of about 2 kg in both barns. In both barns, the concrete floor was littered with wood shavings (600 g/m²) for one trial and straw granulate (1000 g/m²) for two trials. Fresh wood shavings/straw granulate were distributed if necessary. In addition, Miscanthus briquettes (Campus Klein-Altendorf, Universität Bonn, Rheinbach, Germany) were added on both sides of the barns on days 14, 21, and 28 to accommodate pecking behavior. Both barns were equipped with two water lines with drinking nipples, which were shared by nine birds each and four round feeding troughs (1.07 cm space per bird).

**Figure 1:**
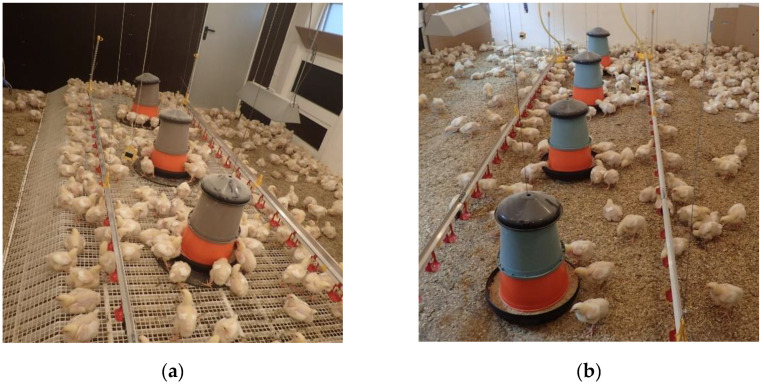
(**a**) Experimental barn with elevated perforated floor and littered areas. (**b**) Control barn with the littered floor.

In one of the barns (experimental barn, Exp), about 50% of the floor space was equipped with an elevated perforated floor (5.3 m × 2.1 m), which was installed in the middle of the barn, under the water lines and feeding troughs at a height of 15 cm (see Figure 1a). The perforated floor was constructed out of plastic elements (Golden Broiler Floor, FIT Farm Innovation Team GmbH, Steinfurt, Germany), measuring 196 cm × 56 cm. The mesh size of the grid was 1.5 cm × 1.5 cm, and the slats were 0.5 cm thick. The residual floor space on the left and the right side of the perforated floor was littered. Adjusted elements connected the littered floor space and the elevated perforated floor to be used as ramps for accessing the floor. The angle between the barn floor and the ramps was about 30°. In the control barn (Con), the animals were kept under approximately commercial conditions on a littered concrete floor (see Figure 1b). 

The broiler chickens of both barns were fed standard broiler feed (Deuka, Deutsche Tiernahrung Cremer GmbH & Co. KG, Düsseldorf, Germany) and water, both were provided ad libitum. In both barns, a negative pressure ventilation system and a climate computer (PL9400, Stienen Bedrijfselektronica B.V., Nederweert, the Netherlands) were used to provide temperature and humidity within the commercial standard for broilers [22]. There was natural light through windows (the size of the windows corresponding to 3% of the floor) and an artificial lighting program with a transition time of 1 h between light and dark periods. The dark period was increased by 2 h per day, from 2 h on the first day of life to 6 h on the third day. It was kept constant at 6 h per day from day 3 to day 29. After day 29, the dark period was decreased by 2 h per day until day 32. The mean light intensity was about 100 Lux in both barns. The broiler chickens were vaccinated against Newcastle disease (day 13), Gumboro (day 18), and infectious bronchitis (day 18).

### 2.2. Behavioral Observations

For behavioral observations, a camera (EQ900F eZ.Hd Series, EverFocus Electronics Corporation, New Taipei City, Taiwan, China) was installed at the ceiling (height: two meters) in the middle of each barn. It was used to film a certain area on the perforated floor and the corresponding area in the control barn. A recorder (AXR-108, Monacor International GmbH & Co. KG, Bremen, Germany) was programmed to record 2 days per week for 24 h. Only daytime videos were analyzed due to the inadequate quality of the videos in the nighttime. One observer conducted the video analysis. To ensure consistent measurements, a subset of 60 scans and ten focal animals was analyzed by a second observer, not involved in the main data analysis, to calculate the inter-observer reliability. Furthermore, intra-observer reliability was calculated, scoring a sample of 60 scans and ten focal animals twice with an offset in time.

#### 2.2.1. Use of the Perforated Floor

To quantify the use of the perforated floor and compare it to the corresponding area in the control barn, the footage was analyzed on two observation days at the beginning (days 2 and 5), the middle (days 16 and 19), and the end (days 26 and 30) of the fattening period of the three trials. Therefore, a screenshot of the videos was taken every 30 min between 6:00 am and 10:30 pm (in total, 1017 pictures for all trials) (scan sampling). A representative area of the perforated floor was defined and marked on the pictures using a digital frame produced with the program GoldenRatio (Markus Welz, Vs. 3.1.4, Krailling, Germany). The width of the area (210 cm) resulted from the width of the perforated floor with ramps in the experimental barn and the length (115 cm) from the distance of five nipples (23 cm between two nipples) of the waterline (in total 2.42 m²). The distance between the nipples was also used to adapt the frame to the corresponding area on the pictures of the control barn. Both areas contained two water lines with five nipples each and one round feeding trough with a diameter of 40 cm, so the usable space was 2.29 m². Birds with more than half of their bodies (including head and tail) within the frame were counted using ImageJ (Wayne Rasband, Vs. 1.51q, National Institutes of Health, Maryland, USA), and the location of each animal was categorized following the definitions in the ethogram in Table 1. All categories were considered as mutually exclusive. The videos were consulted if it was unclear in which direction an animal was holding its head. 

#### 2.2.2. Focal Animal Observations 

The duration of behavior patterns was evaluated by observing focal animals. Again, a frame was added to the footage by using the distance between the nipples of the waterline. The area within the frame measured about 210 cm × 210 cm (in total 4.41 m², usable space 4.28 m²). The experimental barn contained the perforated floor with ramps on both sides, two water lines with ten nipples each and one feeding trough. The corresponding littered area in the control barn included the same resources. On three observation days per fattening period (beginning, day two; middle, day 16 or 19; and end, day 30), ten focal birds per day and barn were selected pseudo-randomly (in total 180 animals for Exp and Con). The first animal, which entered the frame from the left side after 2:00 pm, was observed continuously for two hours or until it left the frame. Then, the video was rewound to the moment the animal entered the frame, and the next animal, which entered the frame from the right side, was chosen for observation. This was repeated until ten birds were observed. To avoid observing an animal twice, the side for choosing the next animal was alternated. The observation started at 2:00 pm because no external disturbances occurred for at least 2 h at this time of the day. While observing the focal birds, their behavior patterns were categorized using INTERACT (Mangold International GmbH, Vs. 17.1, Arnstorf, Germany). The activities defined in the ethogram in Table 1 were differentiated. All categories were considered as mutually exclusive. Based on Norring et al. (2016), the activity was considered to have ended if the animal stopped the activity for 3 s. 

### 2.3. Statistical Analyses

Statistical analysis was conducted using the SAS software (Statistical Analysis Institute, Vs. 9.4, Cary, NC, USA). The observer reliability was calculated for each location separately for the scan sampling and for each behavior in summary for the focal animal observation using Krippendorff’s alpha [24]. The data type was set to metric for each parameter; the number of bootstraps was set to 2000. The classifications suggested by Landis and Koch [25] were used to evaluate reliability (<0.00 = poor, 0.00–0.20 = slight, 0.21–0.40 = fair, 0.41–0.60 = moderate, 0.61–0.8 = substantial, 0.81–1.00 = almost perfect). For descriptive analysis, the mean values and standard deviations (±) were calculated for all parameters.

Residuals and data were checked for distribution based on normality plots created with the univariate procedure in SAS. Generalized linear mixed models were calculated using the GLIMMIX procedure, defining a normal distribution for the data of the usage of the perforated floor, while the distribution for the parameters of focal bird observations was specified as lognormal. For the usage of the perforated floor, a total number of animals, as well as the percentage of those located at ‘Oth’, ‘NF’, and ‘NW’ in relation to all animals on the respective area, were analyzed. All parameters were analyzed separately, including treatment (experimental group, control group), phase of the fattening period (beginning, middle, end), and the interaction between both as fixed effects. The hierarchical structure of the data was considered in the random statement by nesting each screenshot on the day of observation, while the day of observation was nested in the respective trial.

Data analyzed for the focal bird observation were ‘Loc’, ‘Sit’, ‘NF’, and ‘NW’. Here, again, all parameters were analyzed separately, including the fixed factors as described for the usage of the perforated floor. Again, the hierarchical structure of data was accounted for in the random statement, nesting the observed animal on the observation day; these, in turn, were nested in the respective trial. Furthermore, the effects of the abovementioned fixed factors on the duration of each behavioral event of the behavior ‘Sitting inactive’ was analyzed, again including the hierarchical structure in the random statement. Moreover, repeated measures in different animals were accounted for in the random statement. Pairwise comparisons were made using Tukey–Kramer tests. The level of significance was set for *p* < 0.05. A level of *p* < 0.1 was regarded as a tendency.

## 3. Results

For the scan sampling, intra-observer reliability was found to be ‘almost perfect’ for all locations. Inter-observer reliability was ‘moderate’ for the location Oth, ‘substantial’ for the location NF, and ‘almost perfect’ for the location NW. For the focal animal observation, the measurement resulted in ‘almost perfect’ for the intra-observer and ‘substantial’ for the inter-observer reliability. The values of Krippendorff’s alpha coefficients for the different behaviors/locations are presented in Table 2.

### 3.1. Use of the Perforated Floor

**Figure 2:**
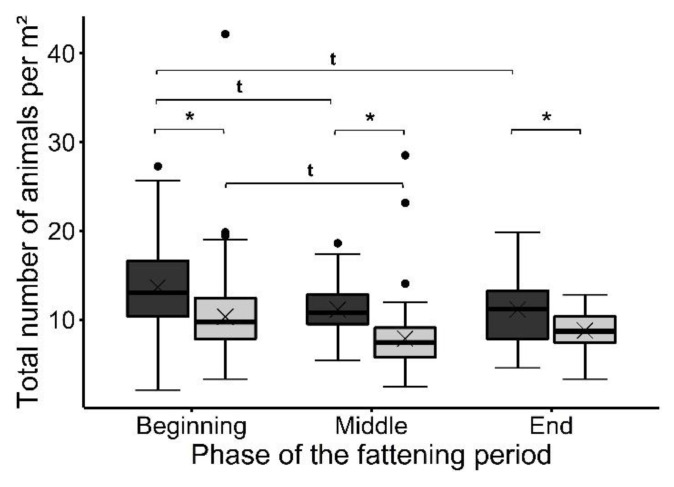
The total number of animals per m² in the observed area at the different phases of the fattening period (*n* = three trials each). Results are presented as boxplots (data range, median, and lower and upper quartile; outliers are included in the graph as dots and means as a cross). Results of the experimental group (Exp) are presented in dark gray; the control group (Con) results are presented in light gray. * Significant difference at *p* < 0.05, ^t^ tendency at *p* < 0.1.

The treatment had a significant effect on the total number of animals in the observed area (*F*_(1, 505)_ = 232.76, *p* < 0.001), with more birds per square meter in the Exp (12.83 ± 4.24) than in the Con (9.67 ± 3.58). A tendency was found for the phase of the fattening period to affect the total number of birds (*F*_(2, 505)_ = 2.55, *p* < 0.1), with more animals counted at the beginning of the fattening period than in the middle (*t* = |2.04|, *p* < 0.1) and the end (*t* = |1.77|, *p* < 0.1). Further, the effect of the interaction between treatment and phase was found not to be significant but revealed a tendency (*F*_(2, 505)_ = 2.63, *p* < 0.1). As shown in Figure 2, there were significantly more birds per square meter in the Exp than in the Con at the beginning (Exp: 14.41 ± 4.69, Con: 10.91 ± 4.37(*t* = |10.89|, *p* < 0.001)), the middle (Exp: 11.76 ± 2.70, Con: 8.28 ± 3.25 (*t* = |8.67|, *p* < 0.001)), and at the end (Exp: 11.77 ± 4.06, Con: 9.26 ± 1.96 (*t* = |7.17|, *p* < 0.001)). There were no significant differences between the phases within the treatment groups. However, a tendency was found for the Exp group, with a higher total number of animals per square meter at the beginning than in the middle (*t* = |2.02|, *p* < 0.1) and at the end (*t* = |2.15|, *p* < 0.1). In the Con group, we detected a tendency for more animals per square meter at the beginning than in the middle of the fattening period (*t* = |1.99|, *p* < 0.1).

The proportion of observed birds in the Exp and Con group located in the different areas and the effects of treatment and phase of the fattening period are summarized in Table 3. The percentage of animals located at Oth differed significantly between the treatment groups (*F*_(1, 505)_ = 463.99, *p* < 0.001). In general, a higher percentage of animals were located at Oth in the Exp group than in the Con group (43.54 ± 14.82% vs. 28.56 ± 15.53%). Further, the phase had a significant effect on the proportion of animals being located at Oth (*F*_(2, 505)_ = 3.81, *p* < 0.05), with animals being located at Oth more often at the beginning (42.90 ± 16.05%) than at the end of the fattening period (28.94 ± 15.02%; *t* = |2.73|, *p* < 0.05). The interaction between treatment and phase also had a significant effect on the proportion of animals being located at Oth (*F*_(2, 505)_ = 32.04, *p* < 0.001). In the Exp group, we detected a higher percentage of animals being located at Oth than in the Con group during all phases (beginning *t* = |7.59|, *p* < 0.001; middle *t* = |16.30|, *p* < 0.001; end *t* = |12.62|, *p* < 0.001). Within the Exp group, there was a tendency for a higher proportion of animals being located at Oth at the beginning than at the end of the fattening period (*t* = |1.93|, *p* < 0.1). In the Con group, the proportion of animals being located at Oth was significantly different between the beginning and middle of the fattening period (*t* = |2.98|, *p* < 0.05) and beginning and end (*t* = |3.43|, *p* < 0.01). In both cases, a higher percentage of broilers were located at Oth at the beginning.

Treatment had a significant effect on the proportion of animals located NF (*F*_(1, 505)_ = 105.01, *p* < 0.001). NF was found in a higher proportion of animals in the Con group (32.77 ± 12.21%) than in the Exp group (26.63 ± 11.12%). A tendency was found for the effect of phase (*F*_(2, 505)_ = 2.82, *p* < 0.1), with a higher amount of animals located NF at the beginning than at the end of the fattening period (*t* = |1.93|, *p* < 0.1).

NW was found to be affected by the treatment (*F*_(1, 505)_ = 196.36, *p* < 0.001), the phase (*F*_(2, 505)_ = 4.09, *p* < 0.05), and the interaction between both (*F*_(2, 505)_ = 25.36, *p* < 0.001). This resulted in a lower percentage of animals located NW at the beginning of the fattening period (31.10 ± 11.77%) than at the middle (35.53 ± 13.62%; *t* = |1.95|, *p* < 0.1) and the end (37.00 ± 11.99%; *t* = |2.74|, *p* < 0.05). Further, a higher percentage of broilers was observed NW in the Con group (38.67 ± 12.82%) than in the Exp group (29.83 ± 10.73%). This pattern was found within every phase of the fattening period (beginning *t* = |3.74|, *p* < 0.01; middle *t* = |12.14|, *p* < 0.001; end *t* = |7.56|, *p* < 0.001). In the Con group, the percentage of animals located NW was lower at the beginning of the fattening period than in the middle (*t* = |4.35|, *p* < 0.001) and the end (*t* = |3.65|, *p* < 0.01). A tendency was found within the Exp group, with a higher proportion of animals located NW at the end than at the beginning (*t* = |1.93|, *p* < 0.1).

### 3.2. Focal Animal Observations

**Figure 3:**
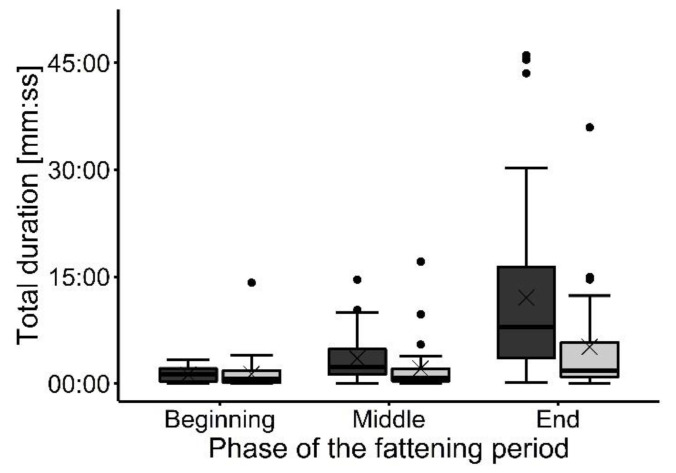
Total time completely observed focal animals (*n* = 80) spent in the observed area compared to runaways (*n* = 100) at the different phases of the fattening period. Results are presented as boxplots (data range, median, and lower and upper quartile; outliers are included in the graph as dots and means as a cross). Completely observed animals are presented in dark gray; runaways in light gray.

Since the observation area did not cover the entire perforated floor, focal birds could leave the frame while not leaving the perforated floor. Therefore, these birds were named ‘runaways’ based on Norring et al. (2016) and were excluded from further analysis in both treatment groups (Exp *n* = 56, Con *n* = 44). This resulted in 34 completely observed focal birds on the perforated floor and 46 in the littered control area. The duration of the runaways observed in the defined area compared to the completely observed animals is shown in Figure 3.

**Figure 4:**
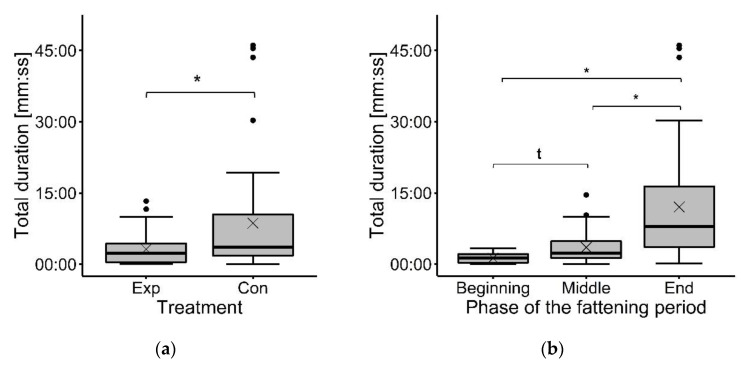
Total time focal animals spent in the observed area: (**a**) in the experimental group (Exp) and the control group (Con); (**b**) in the different phases of the fattening period. Results are presented as boxplots (data range, median, and lower and upper quartile; outliers are included in the graph as dots and means as a cross). * Significant difference at *p* < 0.05, ^t^ tendency at *p* < 0.1.

The treatment had a significant effect on the time the focal animals spent in total in the observed area (*F*_(1, 15)_ = 6.85, *p* < 0.05). More specifically, in the Con group, the animals stayed longer in the observed area than in the Exp group (Exp: 03:09 ± 03:27, Con: 08:39 ± 11:41 (mm:ss)) (see Figure 4a). The total time animals spent in the observed area was found to be affected by the phase of the fattening period (*F*_(2, 15)_ = 11.47, *p* < 0.001). The duration increased during the fattening period, with a higher total time at the end than in the middle (*t* = |3.06|, *p* < 0.05) and at the beginning (*t* = |4.64|, *p* < 0.001) and a higher total in the middle than at the beginning (*t* = |1.94|, *p* < 0.1) (Figure 4b).

**Figure 5:**
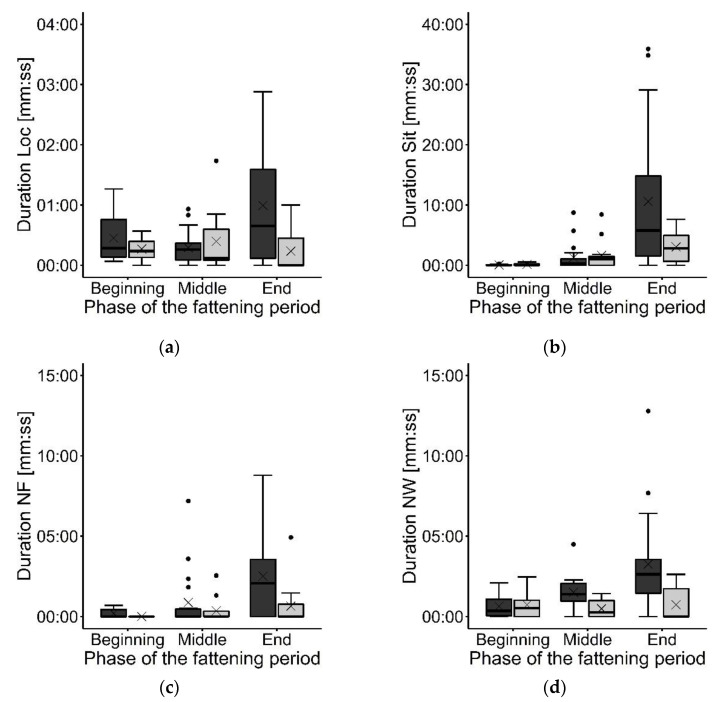
Duration of the different behaviors at the different phases of the fattening period: (**a**) locomotion (Loc); (**b**) sitting inactive (Sit); (**c**) located near the feeding trough (NF); and (**d**) located near the waterline (NW). Results are presented as boxplots (data range, median, and lower and upper quartile; outliers are included in the graph as dots and means as a cross). Results of the experimental group (Exp) are presented in dark gray; the control group (Con) results are presented in light gray.

While 16.3% of the observed animals never showed the behavior Loc, 32.5% never showed Sit. Further, 63.8% of the focal animals had never been located NF, and 26.3% were never observed NW. The treatment had no significant effect on the mean durations of Loc, Sit, NF, and NW (all *F* > 0.00, all *p* > 0.05) (see Figure 5). While Loc, NF, and NW were not affected by the phase (all *F* > 0.81, all *p* > 0.05), an effect of phase was found on the behavior Sit (*F*_(2, 7)_ = 2.28, *p* < 0.05). Pairwise comparisons revealed a longer duration of the behavior Sit at the end (07:25 ± 09:56 (mm:ss)) of the fattening period than at the beginning (00:06 ± 00:10 (mm:ss); *t* = |4.09|, *p* < 0.05) and in the middle (01:25 ± 02:23, (mm:ss); *t* = |2.26|, *p* < 0.1) and a longer duration in the middle than at the beginning (*t* = |2.47|, *p* < 0.05). There was no significant interaction between treatment and phase for the behaviors Loc, Sit, NF, and NW (all *F* > 0.13, all *p* > 0.05).

**Figure 6:**
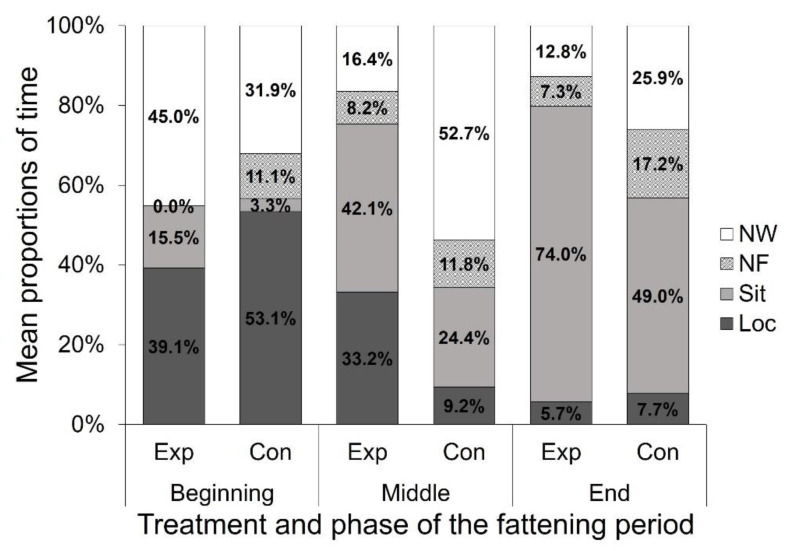
Mean proportions (%) of the behaviors Loc (locomotion), Sit (sitting inactive), NF (located near the feeding trough), and NW (located near the waterline) of the total time focal animals spent in the observed area at the different phases of the fattening period on the perforated floor (Exp) and in the control area (Con).

The mean proportions of the different behaviors of the total time spent in the observed area at the different phases of the fattening period in Exp and Con are shown in Figure 6. Generally, the proportion of the behavior Loc decreased over the fattening period, while the proportion of Sit increased in both treatment groups. 

Further, the duration of each behavioral event of the behavior ‘Sitting inactive’ (Sitting inactive bout, *n* = 184) of 53 different focal animals was analyzed. While the treatment had no significant effect on the duration of the single sitting inactive bouts (*F*_(1, 137)_ = 0.34, *p* > 0.05), the phase had a significant effect (*F*_(2, 137)_ = 5.10, *p* < 0.01). This resulted in longer sitting inactive bouts in the middle (01:15 ± 01:43 (mm:ss); *t* = |2.81|, *p* < 0.05) and at the end of the fattening period (01:38 ± 02:16 (mm:ss); *t* = |3.15|, *p* < 0.01) than at the beginning (00:11 ± 00:10 (mm:ss)). 

## 4. Discussion

The aim of the study was to evaluate the use of an elevated perforated floor equipped with food and water supply by broiler chickens. More animals were observed on the perforated floor than in the littered control area, but focal animals spent less time on the perforated floor compared to the observed littered control area. The animals on the elevated perforated floor were located to a higher proportion at Oth than in the Con. However, there were no differences in the duration of the recorded behaviors. The number of animals in both observed areas decreased, and the duration of the behavior sitting inactive increased over the fattening period. These results show that the broilers’ behavior was not negatively affected by the perforated floor to a high degree.

As expected, there were more animals on the perforated floor than in the littered control area. The 15 cm high floor might be attractive for the animals to perch. Prior studies found that fast-growing broiler chickens are motivated to perch if they are given the opportunity [13,14,15,17]. They prefer elevated platforms to perches, probably due to an increasing body weight, which leads to difficulties finding balance on a perch [14,15]. In accordance with the present results, previous studies have demonstrated that broilers had no difficulties using the elevated floor at the height of 10–50 cm and ramps at an angle of 15°–35° [13,14,15,17].

The mean number of animals on the elevated floor was 12.8 birds/m², which is in accordance with Norring et al. [13] and Bailie et al. [14]. In contrast to these prior studies, in this study, the food and water supply was located on the perforated floor, which forced the animals to use the floor. The fact that the animals in the Exp barn did not have the choice of using the perforated floor should always be taken into account when interpreting the results of this study. In the scan sampling, a higher proportion of animals was categorized as located at ‘other’ on the perforated floor than in the control area. In addition, a higher percentage of broilers was ‘located near the feeding trough’ and ‘located near the water line’ in the littered control area. These findings indicate a usage of the elevated platform apart from using the resources.

Contrary to expectations, the focal birds in the Con stayed longer in the observed area than the animals on the perforated floor. A possible explanation could be that the climate on the perforated floor was uncomfortable for the animals. The manure under the perforated floor led to a higher amount of ammonia in the Exp barn, evaluated in the same experimental setting by Adler et al. [20]. This may have resulted in shorter times spent on the perforated floor. To avoid the problem of high ammonia concentration in barns with perforated floors, installing a manure belt under the elevated perforated floor could be an option and should be further investigated [20].

Prior studies found increased activity when elevated structures were added to experimental pens [4,5]. In addition, we suggested the perforated floor would also be attractive for resting [26]. Therefore, we expected longer durations of either Loc or Sit in the Exp than in the Con. However, we found no differences in the duration of the recorded behavior patterns of the focal animals in both treatment groups. 

In the duration of the single sitting inactive bouts, no differences occurred between Exp and Con. This is contrary to studies by Yngvesson et al. [27] and Forslind et al. [26]. Both studies found that elevated structures increased the duration of resting bouts during the daytime because there was less disturbance by other animals. The results in the current study may be explained by the location of the resources in the observed area. Broilers searching for food and water may have disturbed sitting inactive animals in our study.

In general, the results of the focal animal observations need to be interpreted with caution due to the small sample size after excluding the ‘runaways’. Further, the observation always began at 2 pm and ended at 4 pm at the latest. The behavior of broiler chickens is highly related to the light intensity and differs depending on the time of day [28,29]. In addition, the use of elevated structures may also vary depending on the time of day [5,15]. The results of the focal animal observation only represent the behavior in a narrow time slot. Another limitation of the focal animal observation is that animals categorized as ‘located near the feeding trough’ or ‘located near the waterline’ could also merely walk or sit near the feeding trough or waterline; accordingly, such animals would not be recorded as ‘locomotion’ or ‘sitting inactive’. These variables are not completely independent. Nevertheless, the measured proportions of the different behavior patterns at the total time the animals spent in the observed area were, in general, consistent with prior studies [29,30].

Regarding the phase of the fattening period, the results of the focal animal observations showed that the animals spent in total more time in the observed area, spent more time ‘sitting inactive’, and showed longer sitting inactive bouts at the end of the fattening period than at the beginning. This is consistent with other studies and is suggested to be due to a decrease in the activity of broiler chickens with age [29,30,31]. This is presumably due to higher body weight and an increasing rate of lameness [32] and, therefore, discomfort or pain [33].

As expected, we found slightly more animals per square meter at the beginning of the fattening period than at the end in both treatment groups. In case of the observed area on the elevated perforated floor, this effect could be due to broiler chickens’ decreased walking ability with age and, therefore, difficulties walking up the ramp [5,14]. Another explanation for finding the effect in both treatment groups is an increase in body size with age [34]. 

## 5. Conclusions

To conclude, this study has identified differences in the behavior of broilers when comparing an elevated perforated floor equipped with food and water supply to a control area. There were more animals per square meter on the elevated floor, with a higher proportion of animals located at ‘other’, which implies that the birds used the perforated floor beyond simply using the resources. However, the study did not find differences between the durations of behaviors when comparing the treatment groups. These findings suggest that, in general, the elevated perforated floor is attractive for the animals. However, it does not promote one of the recorded behavior patterns. Further research is necessary to find the optimum design for elevated platforms for fast-growing broiler chickens. A study design with perforated elevated platforms away from feed and water supply would allow a true choice for the animals of opting to be on a certain substrate. It would describe more accurately the physical effort animals invest to require access. Nevertheless, our results show that a partially perforated flooring system could be an option for structuring broiler barns. 

## Figures and Tables

**Table 1:** Ethogram used in the scan sampling and in the focal animal observation.

Method	Behavior/Location	Description
Scan sampling and focal animals	Located near the feeding trough (NF)	The bird is located with more than a half of its body within a radius of one animal’s length around the feeding trough and is holding its head in the direction of the feeding trough (regardless of whether upright position or sitting)
Scan sampling and focal animals	Located near the waterline (NW)	The bird is located with more than a half of its body within a radius of one animal’s length around the water line and is holding its head in the direction of a nipple (regardless of whether upright position or sitting)
Scan sampling	Other (Oth)	All animals not ‘located near the feeding trough’ or ‘located near the waterline’
Focal animals	Locomotion ^1^ (Loc)	The bird is standing or walking (upright position) and is not ‘located near the feeding trough’ or ‘located near the waterline’
Focal animals	Sitting inactive^1^ (Sit)	The bird is resting (sitting with head under the wing or resting on the ground) or lying (the bird is lying on one side with a leg and/or wing stretched out) and is not ‘located near the feeding trough’ or ‘located near the waterline’

^1^ Adapted from Baxter et al. [23].

**Table 2:** Observer reliabilities for scan sampling (*n* = 60 scans) and the focal animal observation (*n* = 10 animals).

Parameter		Krippendorff’s Alpha Intra-Observer	Krippendorff’s Alpha Inter-Observer
Scan sampling:	Oth	0.84	0.42
	NF	0.83	0.75
	NW	0.90	0.85
Focal animal observation:		0.99	0.75

**Table 3:** Mean proportion (%) of animals categorized as located at ‘other’ (Oth), ‘located near the feeding trough’ (NF), and ‘located near the water line’ (NW) at the different phases of the fattening period on the perforated floor (Exp) and in the control area (Con) (*n* = three trials each). Standard deviations are presented in parentheses, n.s. = result is not significant at *p* < 0.05.

Location	Treatment	Phase of the Fattening Period			Treatment *p*	Phase *p*	Treatment × Phase
		Beginning	Middle	End			*p*
Oth	Exp	47.29 (16.66)	46.52 (12.24)	36.86 (11.77)	<0.001	<0.05	<0.001
	Con	38.51 (14.16)	23.06 (10.89)	21.01 (13.69)			
NF	Exp	23.62 (12.71)	26.12 (7.06)	30.57 (10.46)	<0.001	<0.1	n.s.
	Con	28.37 (11.27)	33.29 (10.20)	37.56 (12.83)			
NW	Exp	29.08 (11.63)	27.36 (10.09)	32.57 (9.47)	<0.001	<0.05	<0.001
	Con	33.11 (11.59)	43.65 (11.70)	41.42 (12.62)			

## Data Availability

The data presented in this study are available on request from the corresponding author.
